# Evolutionary and ecological correlates of thiaminase in fishes

**DOI:** 10.1038/s41598-023-44654-x

**Published:** 2023-10-24

**Authors:** Freya E. Rowland, Catherine A. Richter, Donald E. Tillitt, David M. Walters

**Affiliations:** grid.2865.9 0000000121546924 U.S. Geological Survey, Columbia Environmental Research Center, 4200 New Haven Rd, Columbia, MO 65201 USA

**Keywords:** Ichthyology, Ecology, Evolution

## Abstract

Thiamine (vitamin B_1_) is required by all living organisms in multiple metabolic pathways. It is scarce in natural systems, and deficiency can lead to reproductive failure, neurological issues, and death. One major cause of thiamine deficiency is an overreliance on diet items containing the enzyme thiaminase. Thiaminase activity has been noted in many prey fishes and linked to cohort failure in salmonid predators that eat prey fish with thiaminase activity, yet it is generally unknown whether evolutionary history, fish traits, and/or environmental conditions lead to production of thiaminase. We conducted literature and GenBank BLAST sequence searches to collect thiaminase activity data and sequence homology data in expressed protein sequences for 300 freshwater and marine fishes. We then tested whether presence or absence of thiaminase could be predicted by evolutionary relationships, trophic level, omega-3 fatty acid concentrations, habitat, climate, invasive potential, and body size. There was no evolutionary relationship with thiaminase activity. It first appears in Class Actinoptergyii (bony ray-finned fishes) and is present across the entire Actinoptergyii phylogeny in both primitive and derived fish orders. Instead, ecological factors explained the most variation in thiaminase: fishes were more likely to express thiaminase if they fed closer to the base of the food web, were high in polyunsaturated fatty acids, lived in freshwater, and were from tropical climates. These data provide a foundation for understanding sources of thiaminase leading to thiamine deficiency in fisheries and other organisms, including humans that eat uncooked fish.

## Introduction

Thiamine (vitamin B_1_) is an essential cofactor in multiple enzyme complexes required for metabolism of carbohydrates and amino acids^1^. Yet despite being necessary for all life, animals cannot synthesize thiamine de novo, and so the majority must obtain it through diet or direct uptake in the case of fry^2–5^. Biological thiamine synthesis is energetically expensive and complicated^6, 7^. Thiamine in aquatic systems is present at extremely low (picomolar) concentrations; spatially heterogenous; degrades rapidly in the presence of UV^8^, alkaline conditions^9^, and high temperatures^10^; and tends to be rapidly taken up after synthesis^11–13^. Furthermore, having too little thiamine leads to a suite of cardiovascular and neurological issues in humans^3^, foxes^14^, fishes^15^, and other wildlife^16^. Although some effects of thiamine deficiency are reversible, early life deficiency can cause death^3, 17^, and permanent brain damage has been documented in humans^18^. The long-term effects of temporary or intermittent thiamine deficiency in fishes^17^ and the reasons thiamine deficiency complex is showing up more in wild populations^19^ remain poorly understood, in part because thiamine supplementation is inexpensive and easily applied in managed populations.

There are two demonstrated mechanisms for aquatic animals to become thiamine deficient: through a diet that lacks enough thiamine^16^ (e.g., due to poor absorption or lacking nutrients) or through eating something that destroys thiamine before it can be absorbed^17, 20, 21^. Previous research has hypothesized that a diet of lipid-rich prey can lead to thiamine deficiency in fish predators, but these correlative studies have not considered if lipid-rich forage fish also contain thiaminase^22^. Early life stage mortality and sublethal effects in salmonids is linked with low egg thiamine concentrations caused by elevated thiaminolytic enzymes (i.e., thiaminase) present in the maternal diet^21, 23, 24^. Fishes differ drastically in their thiaminase activity^25–27^, but the sources and reasons for thiaminase production are relatively unknown. Bacteria can use thiaminase as a salvage pathway for thiamine biosynthesis^28^, and originally thiaminase activity in forage fishes was thought to be linked to thiaminase-producing bacteria such as *Paenibacillus thiaminolyticus* that had been isolated from Alewife (*Alosa pseudoharengus*)^29^. However, later research revealed no relationship between thiaminase activity and either the amount of *P. thiaminolyticus* thiaminase I protein or the abundance of *P. thiaminolyticus* cells^30^. More recently, Richter et al.^31^ provided evidence for de novo production of thiaminase I by fish (Zebrafish, *Danio rerio*) and identified the genetic basis for thiaminase production in fishes.

The question remains *why* fishes produce thiaminase if it destroys an essential nutrient that fish cannot synthesize? It is highly unlikely that thiaminase production is a prey response to limit predators for two reasons: (1) predators develop TDC when a large portion of their diet has thiaminase, but it can take years^21^; and (2) this feedback loop is too slow to benefit prey producing thiaminase who might have two or three generations until predator populations begin to experience reproductive failure. One possible explanation of thiaminase production is the result of strong selective pressure as a method to partially resynthesize thiamine^6^. Fish that express thiaminase activity are not themselves deficient in thiamine^26^, suggesting that mechanisms exist to partition thiaminase activity from thiamine within the tissues of fish that express thiaminase. However, the mechanisms, efficiency, and energetic requirements of thiaminase partitioning have not been elucidated. Thiaminase activity has been found to increase with disease challenges^32^ and with diet quality and stress^33^. However, not all fishes produce thiaminase, and thiaminase activity levels are highly variable^26, 33, 34^. The evolutionary history of thiaminase production in fishes is also unknown. Thiaminase may be more common in primitive than derived North American freshwater fishes^34, 35^, but these studies did not consider marine species.

We sought to explore whether evolutionary and ecological characteristics could explain thiaminase presence in fishes. Specifically, we evaluated: (1) if phylogenetic relationships would predict thiaminase presence or activity, consistent with previous work on North American freshwater fishes^34, 35^; and (2) if ecological or physiological characteristics of trophic level, habitat use during foraging, salinity tolerance, or lipid content predicted thiaminase presence or activity in fishes. Associations of thiaminase with these ecological or physiological factors would aid in evaluation of risk for thiaminase-induced TDC in piscivorous fishes, wildlife, and humans.

## Methods

### Data collection

Data on thiaminase I activity of 300 fishes were compiled from existing literature^25–27, 32–55^ and a GenBank search for protein sequences coded by expressed transcripts with significant homology to Zebrafish (*Danio rerio*) thiaminase I (Richter et al. 2023; GenBank accession number NP_001314821.1). The thiaminase I enzyme of Zebrafish is homologous to a candidate thiaminase gene identified in Alewife. The empirically derived mass and isoelectric point of the thiaminase I activity extracted from Alewife tissues exactly matched that predicted for the candidate Alewife thiaminase I gene^31^. We limited the search to fish species with at least 10,000 predicted protein sequences in GenBank. We conducted a protein BLAST sequence search for each of the species against the Zebrafish thiaminase predicted protein sequence^31^. Fishes were scored thiaminase positive if they had an expressed predicted protein sequence with at least 35% sequence identity^56^ to Zebrafish thiaminase and contained the predicted active site cysteine (C153 in NP_001314821.1).

The BLAST search and literature agreed for 23 fishes where both genetic and literature thiaminase data were available. There were few disagreements. Sea Lamprey (*Petromyzon marinus*) were categorized as thiaminase positive in the literature^36^ but negative in BLAST. Since Boggs et al. (2019) suggested little to no thiaminase activity for another lamprey species and the BLAST data are more comprehensive, we categorized them as negative. Ninespine Stickleback (*Pungitius pungitius*) was listed as thiaminase positive in Riley and Evans^35^ based on activity of 85 ± 60 pmol/g/min^26^. However, the BLAST data indicated it was thiaminase negative and the high standard deviation in measurements suggested many low values. Furthermore, a more recent study showed very little thiaminase activity^57^. Thus, we categorized the Ninespine Stickleback as thiaminase negative. Lake Whitefish (*Coregonus clupeaformis*) was categorized as thiaminase positive based on Deutsch and Hasler^58^ but was BLAST negative. Since we have lower confidence in the sole literature value, we categorized Lake Whitefish as negative. Bowfin (*Amia calva*) was positive in two studies^36, 46^ but also listed as both positive and negative^36^. Thiaminase analysis in these studies was conducted on whole-body Bowfin homogenates, and it is possible that positive results may have resulted from analyzing Bowfin that contained thiaminase-rich prey in their guts, so they were eliminated from analysis. European Perch (*Perca fluviatilis*) was positive in one study^48^ but negative in BLAST. We put higher trust in the BLAST data since published work^48^ only reports positive or negative activity and did not report a range of measured values. All entries were checked closely by two separate people for completeness and accuracy.

We obtained family, order, and ecological data from fishbase.org^59^ for all species included in our final thiaminase database. Data on maximum total length (cm), trophic level estimate, and median Omega-3 concentration were treated as continuous variables. A species’ invasive ability (*i.e.*, documented negative ecological impacts in areas where they are introduced or labeled as potential pest species on Fishbase^59^), climate range (i.e., polar, boreal, temperate, subtropical, tropical, deep-water), habitat (benthic, benthopelagic, or pelagic), and whether a fish spends the majority of its life in marine or freshwater environments were treated as categorical variables.

### Data analysis

We analyzed all data in R v4.1.2^60^. We tested for phylogenetic relationships among fish families and presence/absence of thiaminase using a published fish phylogeny including the Classes Sarcopterygii, Chondrichthyes, and Actinopterygii^61^, and among orders using a phylogeny of ray-finned fishes of Class Actinopterygii only^62^. We used the R package *ape*^63^ to prune the tree to fish orders/families where we had data using the ‘drop.tip’ function. We then used ‘make.simmap’ in the *phytools* package^64^ to fit continuous-time reversible Markov models to estimate the evolution of thiaminase at each node for 500 simulations. The models assumed equal (0.5/0.5) root node prior probabilities of presence or absence of thiaminase conditioned on the published fish phylogenies. We used these simulations to estimate the probability that an ancestral state/root node had thiaminase, represented as pie charts at each node.

To explore the ecological determinants of thiaminase activity we used Bayesian binomial models with a logit-link function in the *rstanarm*^65^ R package. We used weakly informative priors with a mean of zero and standard deviation of 2.5. We ran each Bayesian model for 10,000 iterations and discarded the first half as a warm-up to obtain 20,000 simulations for analysis. We confirmed convergence using Gelman–Rubin statistic (R̂ < 1.01)^66^ and by examining trace plots. None of the models had influential outliers as assessed by leave-one-out cross-validation (“loo”) in the *rstan* package^67^. We report the coefficients as the mean and the 95% credible interval (95% CRI), which is the range of values for posterior samples. We computed Bayesian R^2^ for the regression models (i.e., multiple regression, trophic level, and omega-3 models) to explore the proportion of variance explained by the models^68^.

## Results

**Figure 1:**
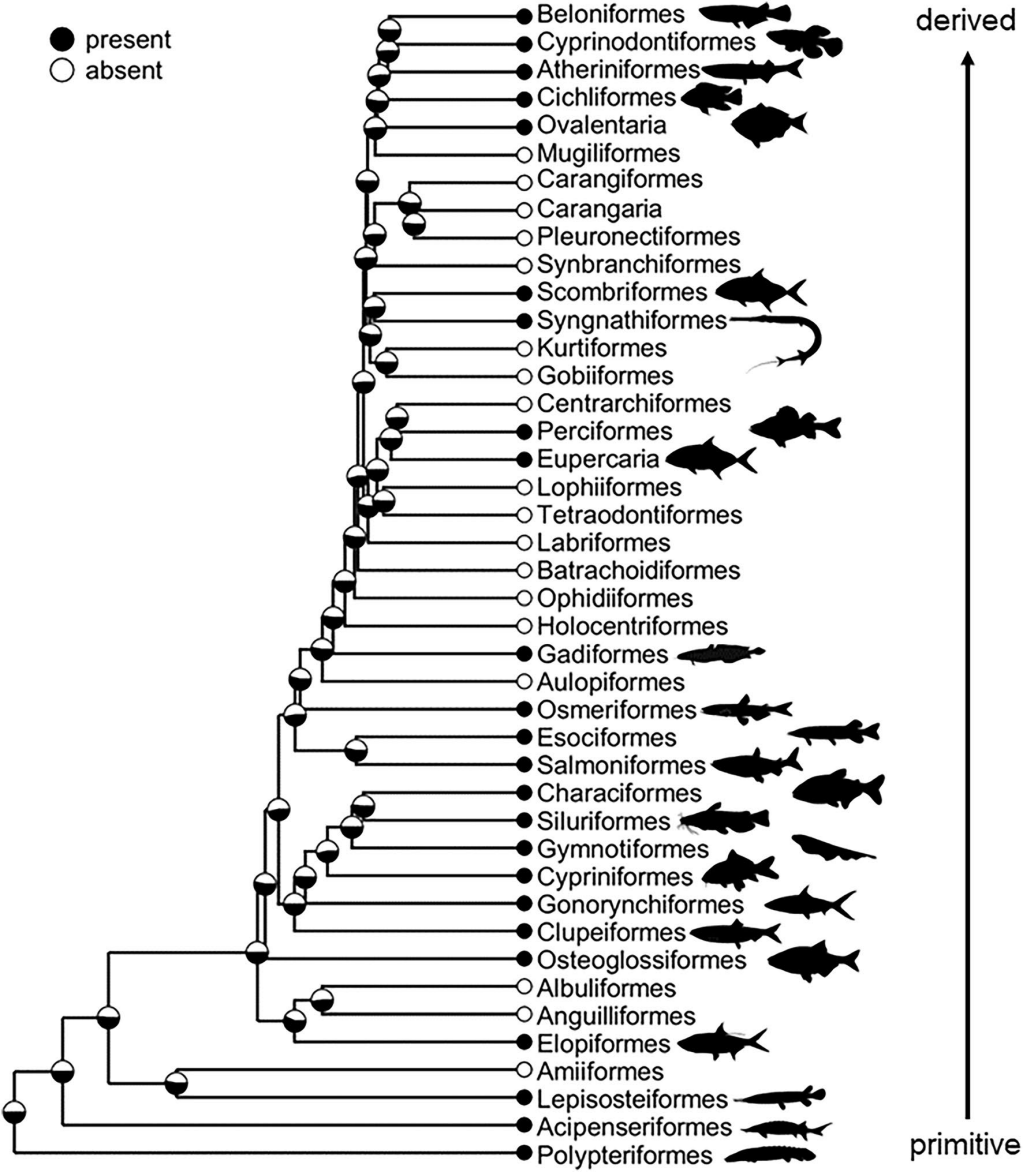
Presence (black) or absence (white) of thiaminase across 42 fish orders within Actinoptergyii that overlap between our data and Rabosky, et al.^62^. If at least one species within the order had  evidence of thiaminase activity, we coded it as having thiaminase present. Branch lengths indicate time since evolution such that longer branches show orders that evolved longer ago. The pie chart at each node is the probability of the ancestral state having thiaminase based on 500 Monte Carlo simulations. Silhouettes represent common body forms within each order (downloaded from phylopic.org; all images public domain/creative commons).

**Figure 2:**
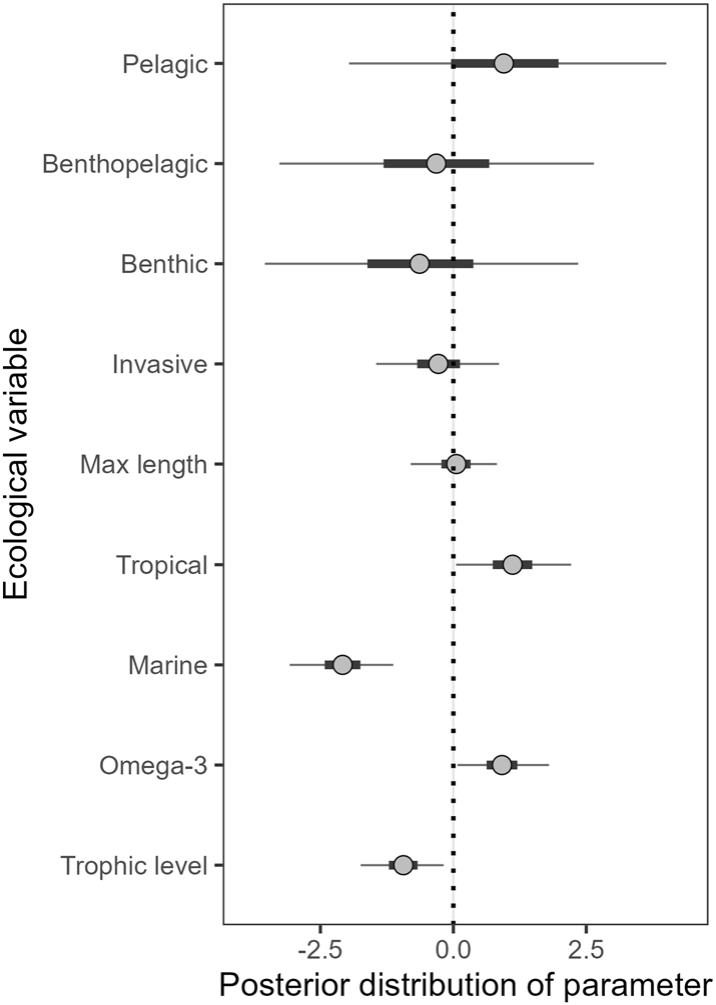
Results from the Bayesian multiple regression of ecological variables vs. thiaminase presence/absence. Trophic level, omega-3, and maximum length (cm) are continuous variables, and the others are categorical (yes = 1, no = 0). The dotted line shows 0, so posterior histograms that do not overlap zero have good evidence of being related to thiaminase (e.g., if most of the posterior is positive, this suggests it is related to thiaminase presence). The 95% credible interval within each histogram is shaded and the median is represented as vertical solid line. Overall, this model explained 36% of the variation in the data.

Our species pool included 300 fishes that were tested for thiaminase activity or were searched for thiaminase proteins in their expressed sequence libraries. Of these, less than half (n = 119) had thiaminase. These species represent a broad range of sizes, climates, and habitats (Supplementary Table S1), with representation from 124 families, 56 orders, and 3 classes of fishes. Despite having broad evolutionary representation, we found no evidence of an evolutionary pattern in thiaminase of fishes (Fig. 1, Supplementary Fig. S1). We found no evidence for thiaminase production in more primitive fishes like Coelacanth (Class Sarcopterygii), lampreys (Class Hyperoartia), or cartilaginous fishes like sharks, rays, and skates (Class Chondrichthyes; Supplementary Fig. S1). Thiaminase first appears in the most primitive ray-finned fishes (Class Actinopterygii) such as the Bichir (Order Polypteriformes), Mississippi Paddlefish (Order Acipenseriformes), and Spotted Gar (Order Lepisosteiformes). Thiaminase is distributed across the entire Actinopterygii phylogeny to more derived orders such as the live-bearing fishes (Order Cyrinidontiformes; Fig. 1). The Markov model simulations indicated equal probability of thiaminase across all nodes of the phylogeny (Fig. 1, Supplementary Fig. S1). Ecological factors explained nearly 40% of the variation in thiaminase presence or absence (Bayesian R^2^ = 0.36, Fig. 2). Trophic level (b_TL_ = − 0.88, 95% CRI [− 1.72, 0.11]; Supplementary Table S2), and association with marine environments (b_marine_ = − 2.01, 95% CRI [− 3.07, − 1.12]) were negatively related to thiaminase presence in fishes (Fig. 2). Habitat (benthic, benthopelagic, or pelagic), invasive potential, and size (as maximum total length) were not predictors of thiaminase (Fig. 2).

**Figure 3:**
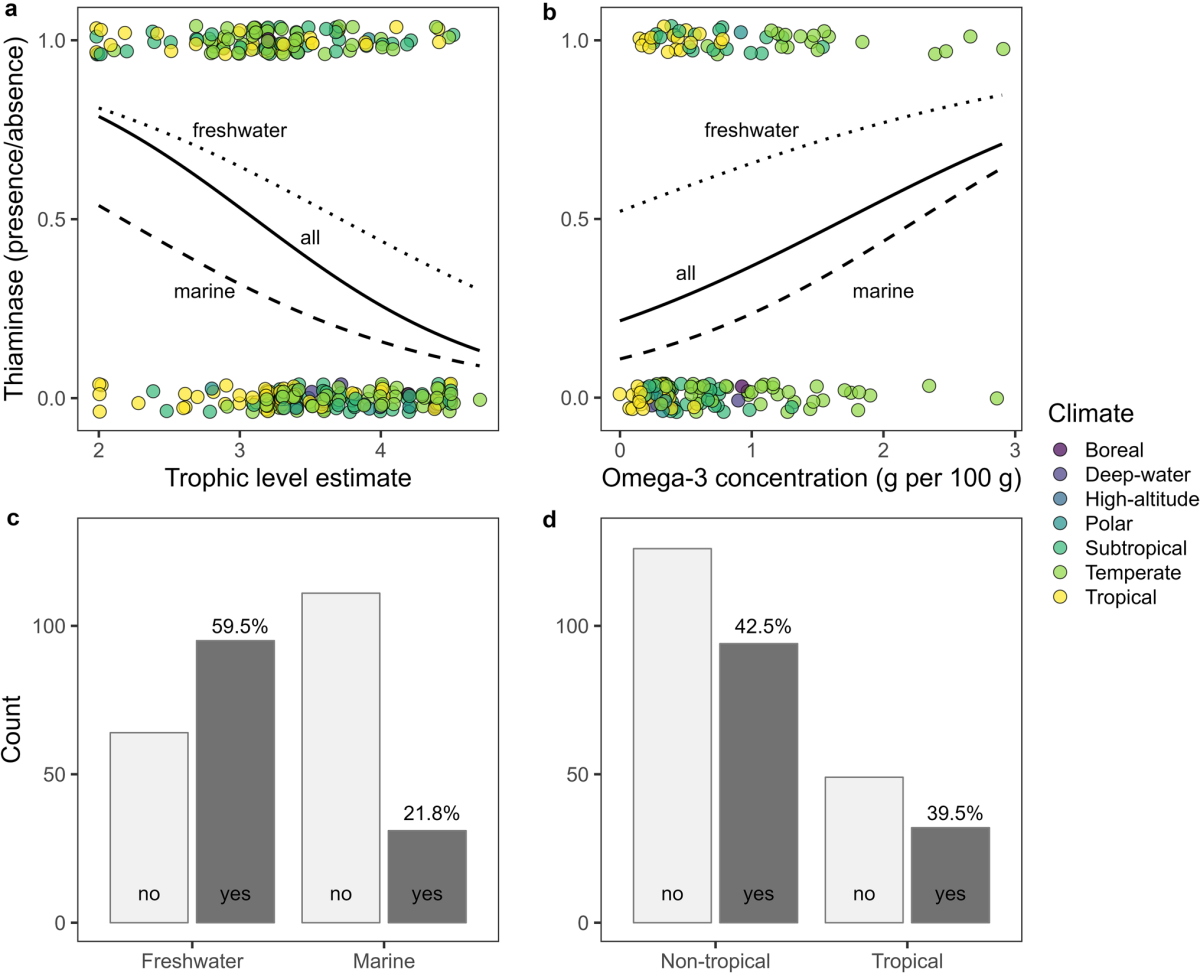
Exploration of coefficients that had the most support for predicting thiaminase in the multiple regression (Fig. 2). Top panels show the continuous relationships between (**a**) trophic level and (**b**) omega-3 concentration and thiaminase activity. Each point represents a fish that either does not have thiaminase (y = 0) or had evidence of thiaminase activity (y = 1) in the literature or BLAST sequence, color coded by a fish’s climate region. Each regression was fit separately with freshwater only (dotted line), marine only (dashed line), or all fishes included (solid line). Bottom panels show the presence (dark bars, labeled as “yes”) or absence (light bars, labeled as “no”) of thiaminase activity separated by (**c**) freshwater vs. marine, and (**d**) non-tropical and tropical. Percentage of fishes with thiaminase within a group is on top of the dark bars.

Probability of thiaminase production decreased as trophic level increased, meaning that lower trophic levels were more likely than top predators to have thiaminase, and the trophic level model alone explained 10% of the variation in the data (Bayesian R^2^ = 0.10, Fig. 3a). Marine species were less likely to have thiaminase (Fig. 3c); only 21.8% of marine fishes compared to 59.5% of freshwater fishes had thiaminase. Two ecological traits increased probability of thiaminase. Omega-3 concentration (b_omega-3_ = 0.94, 95% CRI [0.07, 1.82]; Supplementary Table S3) and tropical climate (b_tropical_ = 1.16, 95% CRI [0.03, 2.23]) were positively related to thiaminase in fishes (Fig. 2). Higher omega-3 concentrations resulted in higher probability of thiaminase production, and omega-3 concentration alone explained 5% of the variation in the data (Bayesian R^2^ = 0.05, Fig. 3b). Tropical species had a nearly equal proportion of thiaminase positive fishes as non-tropical species (Fig. 3d), so the increased probability of thiaminase presence in tropical fishes only appears after trophic level, omega-3 concentrations, and marine/freshwater status are included in the model.

## Discussion

There are two previous studies exploring why thiaminase is present in some fishes but not others^34, 35^, and with adding the current study we still do not know why any fishes make thiaminase. Yet dietary thiaminase has been linked to thiamine deficiency since the 1940s in taxa as diverse as silver foxes^14^, reptiles^69, 70^, fishes^17, 71^, marine mammals^72^, and humans^3^. We found that ecological traits rather than evolutionary patterns explain thiaminase presence among hundreds of fishes. Fishes with lower trophic levels, high polyunsaturated fatty acids, freshwater habitats, and from tropical climates were more likely to produce thiaminase.

### Evolutionary patterns of thiaminase in fishes

Thiaminase is not present in ancient fishes. Lampreys, cartilaginous fishes, and the Coelacanth (*Latimeria chalumnae*) all lack the genes to produce the thiaminase protein. For both family- and order-level analyses, there was no phylogenetic relationship of thiaminase presence/absence. The trait first appears in the Class Actinopterygii (ray-finned fishes) Order Polyteriformes (e.g., bichirs, reedfishes), which evolved 368 million years ago^61^, but the presence/absence of thiaminase has no discernable patterns within Class Actinopterygii. Previous work has reported thiaminase activity and presence were generally higher in basal teleosts (clupeids, cyprinids, and catostomids) than in more derived neoteleosts (e.g., percids and centrarchids)^34, 35^. However, with a tenfold larger data set we were unable to discern any phylogenetic patterns. Certain orders have more members with thiaminase (Clupeiformes and Cypriniformes), suggesting there must be some evolutionary reason for its presence. While other species can and do produce thiaminase, clupeids in particular are well known to cause thiamine deficiency if they are a large portion of predator diets^17, 50, 70, 73–75^. A better understanding of how thiaminase in Clupeiformes such as alewife differs from that in Cypriniformes such as carps and other fishes is needed.

### Ecological patterns of thiaminase in fishes

Ecology appears to be an important determinant of thiaminase presence in fishes. Thiaminase I activity in carp increased in response to pathogenic bacterium exposure, suggesting that thiaminase may be modulated in response to disease challenges^32^. It may be that tropical and freshwater fishes are more likely to have thiaminase because of higher exposure to pathogens. Previous work has found higher disease prevalence for shorebirds occupying tropical freshwater than marine temperate or arctic regions^76^, and viral and bacterial loads are higher in freshwater systems^77^. Moreover, a large-scale metanalysis across taxa found biotic interactions are stronger in the tropics^78^. This suggests that habitat and climate and their influence on exposure to pathogens may be a reason for thiaminase presence.

Trophic level was the strongest predictor of thiaminase among the ecological variables we explored. Very few top trophic level predators (TL > 4) were thiaminase positive. The reasons for fishes producing thiaminase remain unclear. Lower trophic food items like seston and zooplankton are highly variable in thiamine concentrations^79^. Likewise, median thiamine concentrations can dilute with increases in trophic level^80, 81^. There is some evidence that thiaminase production may be related to diet composition^33^, but there are many remaining questions. More research is needed to understand how thiaminase-producing fishes compartmentalize thiamine and thiaminase within their tissues and in identifying the ecological advantages of producing thiaminase.

One of the more interesting results was the strong positive relationship between omega-3 concentration and thiaminase presence, independent from trophic level (omega-3 vs. trophic level: F_1, 154_ = 0.203, *p* = 0.653). Thiamine-deficiency complex in North American fisheries is thought to be the result of thiaminase in prey fishes destroying thiamine in predators gut contents as they pass through the gut^15, 17, 23, 26^. In contrast, thiamine deficiency (called M74) in Atlantic Salmon (*Salmo salar*) from the Baltic Sea has been correlated with consumption of Sprat (*Sprattus sprattus*) and Atlantic Herring (*Clupea harengus*)^50^. In the Baltic, thiamine deficiency is presently thought to be caused by high lipid density leading to low thiamine concentrations per unit energy^22, 82, 83^. However, there has been no reported evidence that oxidative stress from highly unsaturated omega-3 fatty acids results in thiamine deficiency in consumers eating diets without thiaminase. Diets containing both high concentrations of omega-3 fatty acids and thiaminase confound attempts to uncover drivers of thiamine deficiency observed in M74-affected fish. It is also possible forage fish with high lipids^22^ and thiaminase presence such as in Sprat^27^ have additive negative effects on thiamine concentrations in predators. More efforts to disentangle whether thiaminase, high lipids (such as omega-3), or both cause thiamine deficiency are needed to understand threats to fisheries.

### Future directions

A critical step forward in determination of which fish species produce thiaminase will come from our understanding of biological function(s) of thiaminases in fish. Why fishes produce thiaminase remains unknown, but the discussions may have been hindered because of the tendency to focus on thiaminase’s thiamine-degrading properties (and aforementioned impact on predators) rather than its function as a benefit to organisms. In bacteria, thiaminase II hydrolyzes the thiamine break-down product of formylaminopyrimidine (N-formyl-4-amino-5-aminomethyl-2-methylpyrimidine) to 4-amino-5-hydroxymehtyl-2-mehtylpyrimidine (HMP) which is then recycled in a thiamine biosynthetic pathway^7^. More recently, Sannino et al.^28^ demonstrated that the bacterium *Burkholderia thailandensis* uses thiaminase I to salvage precursors from environmentally available thiamine derivatives, and then preferentially uses these precursors for thiamine synthesis. This preference of auxotrophic *B. thailandensis* for thiamine precursors over thiamine itself has also been observed in the abundant SAR11 clade marine bacteria^84^. These mechanisms for salvage of thiamine precursors have not been demonstrated in fishes but offer areas of investigation within the fish microbiome.

Thiaminase I most certainly offers a selective advantage in fishes that possess this gene. Some possible advantages offered by thiaminase production include: (1) aide in thiamine production of commensal bacteria of the microbiome; (2) ecological advantages through population control of predatory species that forage on thiaminase-producing fishes; and (3) enhanced health of thiaminase-producing species of fish through greater immune function. Thiamine is the least metabolically stable B vitamin^85^ due to an oxidative side-reaction that readily damages the thiazole moiety^86^. Thiamine is also degraded in the presence of UV, sulfites, or high pHs, exacerbating its scarcity in natural environments and offering an advantage to organisms with microbiomes containing the ability to resynthesize thiamine from its breakdown products or precursors^2^. It is interesting that thiaminase production is most common in lower trophic levels, meaning that forage fishes are most likely to produce it. Thiaminase I as a thiamine salvage pathway in their microbiome would offer a strong advantage to fishes in environments with low and unstable thiamine supplies. However, it is unlikely that thiaminase I in forage species serves as a selective force to control reproduction and subsequently population size in salmonine predators. Laboratory experiments demonstrated that it took at least two years to induce TDC in eggs and fry of Lake Trout fed a thiaminase-rich diet^21^, a timeline too long for the feedback loop to benefit prey producing thiaminase.

Last, there is evidence that thiaminase I in fishes may be associated with immune function. Thiaminase activity within fishes is found to be greatest in tissues known to have immune function, such as head, kidney, gill, and spleen^44, 47^. Additionally, thiaminase activity increased in carp injected with a pathogenic bacterium (*Aeromonas salmonicida*), suggesting a relationship between thiaminase expression in fish and immune status^32^. Thiaminase I in fishes may have antimicrobial activity, which would be a significant health benefit for survival. The subcellular localization of thiaminase in lysosomes^87^ is consistent with such an antimicrobial activity.

The physiological function of thiaminase I in fishes remains in question at this point. Better understanding of these functions will ultimately help our predictions of ecological determinants for thiaminase production in fishes, as well as evolutionary significance of this fascinating enzyme.

## Conclusions

The present work shows that de novo thiaminase production in fishes is widespread. We found no evolutionary relationship with thiaminase activity. Thiaminase appears in Class Actinoptergyii and is present across the entire phylogeny in both primitive and derived fish orders within this Class. Computer simulation resulted in the probability of all families in the Actinoptergyii Class having thiaminase, suggesting the genes have been widely retained. Ecological factors explained the most (40%) variation in thiaminase; fishes were more likely to express thiaminase if they feed closer to the base of the food web, were high in polyunsaturated fatty acids, lived in freshwater, and were from tropical climates.

Determining sources of thiaminase can help predict spatial and temporal patterns of the risks of thiamine deficiency globally. Thiamine deficiency is considered one of the top emerging issues for wildlife^19^. As the climate changes, certain fish communities are shifting their ranges. Species like Northern Anchovy (*Engraulis mordax*, a thiaminase positive fish) have reached record abundances in the Pacific Ocean along the southern portion of the United States^88^, causing thiamine deficiency in Pacific Salmon^74^. Understanding which prey species produce thiaminase, why they produce it, and how prey range and population sizes may change with climate is a necessary foundation for predicting and managing thiamine deficiency in fisheries.

### Supplementary Information

Supplementary Information.

## Data Availability

All data and R code are publicly available on Zenodo (https://doi.org/10.5281/zenodo.8263918).
