# Supplementary material for: Evolutionary and ecological correlates of thiaminase in fishes
Source: Sci Rep. 2023 Oct 24;13:18147. doi: 10.1038/s41598-023-44654-x (PMC10598016; doi:10.1038/s41598-023-44654-x)
Supplement: Supplementary file 1 — Supplementary Information. [file 41598_2023_44654_MOESM1_ESM.docx]

**Supplementary Information**

| Climate | Total fishes | Thiaminase active | Marine | Freshwater | Average maximum length (cm) | Avg Omega-3 (g per 100 g) | Benthic | Bentho-pelagic | Pelagic |
| --- | --- | --- | --- | --- | --- | --- | --- | --- | --- |
| Boreal | 3 | 1 | 2 | 1 | 45 (118) | 0.93 (NA) | 1 | 1 | 1 |
| Deep-water | 5 | 0 | 5 | 0 | 121 (51) | 0.45 (87) | 4 | 1 | 0 |
| High altitude | 1 | 1 | 0 | 1 | 13 (NA) | NA | 0 | 1 | 0 |
| Polar | 7 | 1 | 7 | 0 | 73 (79) | 0.44 (53) | 5 | 1 | 1 |
| Subtropical | 87 | 41 | 44 | 43 | 91 (213) | 0.60 (50) | 40 | 38 | 9 |
| Temperate | 116 | 49 | 53 | 63 | 73 (103) | 1.10 (61) | 60 | 37 | 9 |
| Tropical | 81 | 32 | 31 | 50 | 54 (127) | 0.29 (62) | 35 | 39 | 7 |
| Total | **300** | **125** | **142** | **158** | **73 (165)** | **0.73 (80)** | **145** | **118** | **37** |

**Table S1.** Summary of the ecological information for fishes in the present study. Coefficient of variation (CV) is in parentheses. NA indicates there were not enough data for an estimate.

**Table S2**. Model slope estimates of trophic level vs. thiaminase all data, freshwater species only, and marine species only. Note: coefficients are logit-transformed. ESS = effective sample size

| **Data** | **slope estimate (median)** | **95% credible interval** | **% posterior < 0** | **ESS** |
| --- | --- | --- | --- | --- |
| All (n = 295) | -1.16 | [-1.62, -0.72] | 100% | 13,276 |
| Freshwater (n = 153) | -0.82 | [-1.48, -0.19] | 99.5% | 13,249 |
| Marine (n = 142) | -0.91 | [-1.63, -0.26] | 99.6% | 11,053 |

**Table S3**. Model slope estimates of omega-3 fatty acids (g per 100 g) vs. thiaminase fit with all data (solid line), marine only (dashed line), and freshwater only (dotted line). Note: coefficients are logit-transformed. ESS = Effective sample size

| **Data** | **slope estimate (median)** | **95% credible interval** | **% posterior > 0** | **ESS** |
| --- | --- | --- | --- | --- |
| All (n = 167) | 0.79 | [0.23, 1.36] | 99.7% | 14,587 |
| Freshwater (n = 49) | 0.62 | [-0.52, 1.85] | 85.9% | 12,861 |
| Marine (n = 118) | 0.93 | [ 0.25, 1.65] | 99.7% | 12,099 |


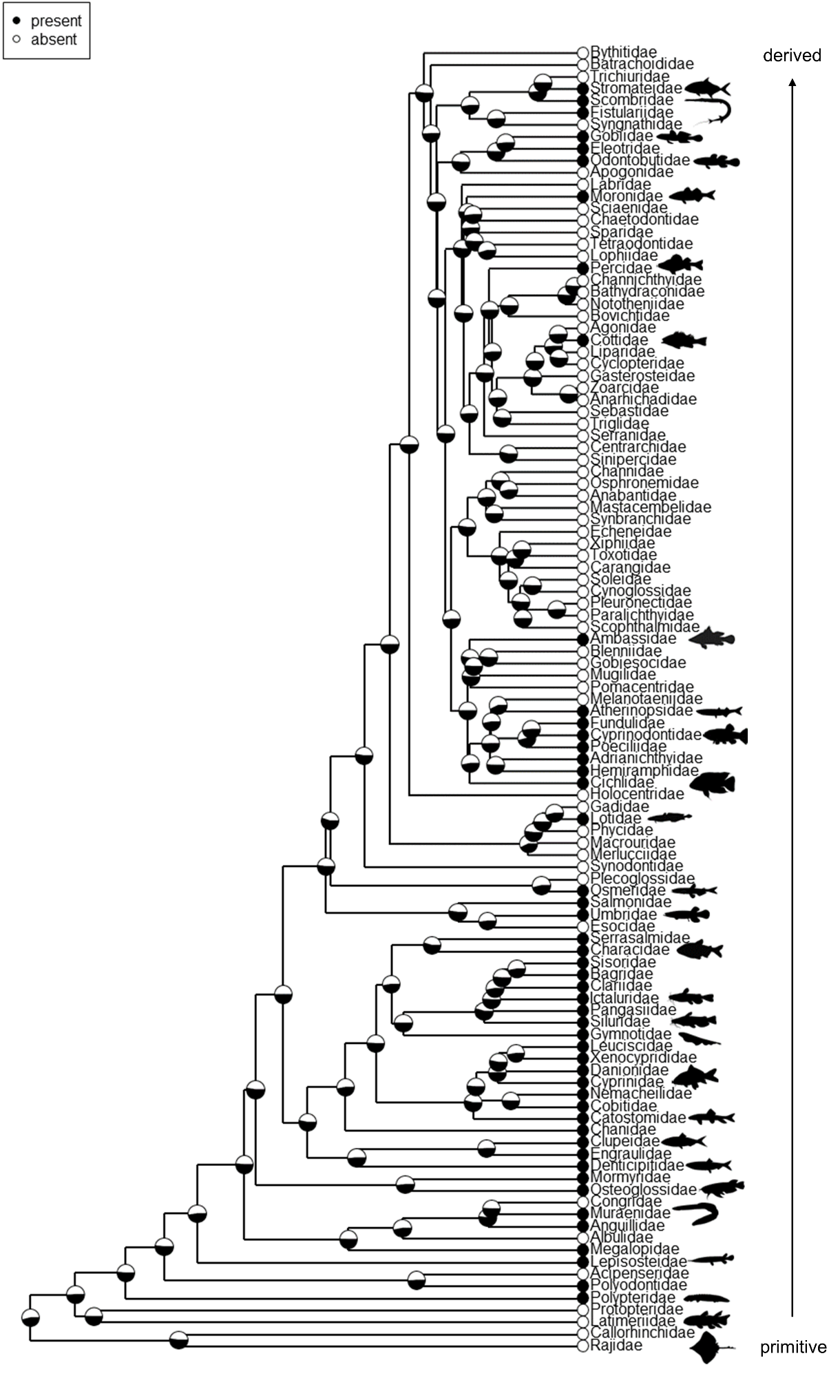


**Fig. S1**. Presence (black) or absence (white) of thiaminase across 124 fish families that overlap between our data and Betancur-R, et al. ^1^. If at least one species within the family had thiaminase, we coded it as having thiaminase present. The pie chart at each node is the probability of the ancestral state having thiaminase based on 500 Monte Carlo simulations.

**References**

1 Betancur-R, R. *et al.* Phylogenetic classification of bony fishes. *BMC Evolutionary Biology* **17**, 162, doi:10.1186/s12862-017-0958-3 (2017).
